# Supplementary material for: Non-linear associations of amyloid-β with resting-state functional networks and their cognitive relevance in a large community-based cohort of cognitively normal older adults
Source: Alzheimers Res Ther. 2026 Mar 12;18:90. doi: 10.1186/s13195-026-01986-w (PMC13097708; doi:10.1186/s13195-026-01986-w)
Supplement: Supplementary file 1 — Supplementary Material 1. [file 13195_2026_1986_MOESM1_ESM.pdf]

Non-linear associations of amyloid-β with resting-state functional networks and their cognitive relevance in a large community-based cohort of cognitively normal older adults

Supplementary Table S1. Demographics, cerebrospinal fluid (CSF), and neuropsychological assessments by CSF biomarker status

	Groups		BM+ vs. BM-		
	BM-	BM+	Statistical test	Test statistic[1]	*P* value
N	837	131			
Age (years)	63.2 [57.6 - 68.6]	68.5 [64.0 - 71.5]	Wilcoxon rank-sum	36080	< 0.001***
Sex (female)	550 (65.7%)	91 (69.5%)	Chi-square	0.714	0.551
Education (years)	16.0 [16.0 - 18.0]	16.0 [16.0 - 18.0]	Wilcoxon rank-sum	55270	0.985
APOE ε4 alleles			Chi-square	87.270	< 0.001***
0	561 (72.9%)	43 (34.4%)			
1	190 (24.7%)	64 (51.2%)			
2	19 (2.5%)	18 (14.4%)			
CSF Aβ (pg/ml)	1272.0 [969.6 - 1637.0]	638.9 [503.1 - 770.8]	GLM (age- and sex-adjusted)	-15.450	< 0.001***
CSF T-tau (pg/ml)	155.9 [123.9 - 199.3]	226.7 [187.1 - 300.6]	GLM (age- and sex-adjusted)	13.300	< 0.001***
CSF P-tau (pg/ml)	13.4 [10.4 - 17.2]	21.3 [17.1 - 27.6]	GLM (age- and sex-adjusted)	15.370	< 0.001***
CSF P-tau/Aβ ratio	0.011 [0.009 - 0.013]	0.031 [0.026 - 0.043]	GLM (age- and sex-adjusted)	30.100	< 0.001***
RCFT immediate free recall	18.5 [14.0 - 23.0]	16.5 [12.5 - 20.2]	GLM (age- and sex-adjusted)	-1.752	0.180
RCFT delayed free recall	18.0 [13.0 - 22.5]	16.0 [11.6 - 20.4]	GLM (age- and sex-adjusted)	-1.531	0.232
Recognition of RCFT elements	21.0 [20.0 - 22.0]	20.0 [19.0 - 22.0]	GLM (age- and sex-adjusted)	-0.238	0.974
RCFT copy accuracy score	33.0 [30.5 - 35.0]	33.0 [31.0 - 34.0]	GLM (age- and sex-adjusted)	0.906	0.548
JoLO	26.0 [23.0 - 28.0]	25.0 [23.0 - 28.0]	GLM (age- and sex-adjusted)	0.078	0.989
RAVLT delayed recall	10.0 [7.0 - 12.0]	9.0 [5.0 - 11.0]	GLM (age- and sex-adjusted)	-1.520	0.232
Letter Fluency (FL)	28.0 [24.0 - 34.0]	30.0 [25.8 - 35.0]	GLM (age- and sex-adjusted)	2.266	0.061
Animal Fluency	21.0 [18.0 - 25.0]	21.0 [18.0 - 25.0]	GLM (age- and sex-adjusted)	1.074	0.463
TMTA	32.0 [26.0 - 39.0]	33.0 [28.0 - 42.0]	GLM (age- and sex-adjusted)	-0.014	0.989
TMTB	64.0 [51.0 - 82.0]	66.0 [57.0 - 95.0]	GLM (age- and sex-adjusted)	0.331	0.953

BM- = biomarker-negative; BM+ = biomarker-positive; APOE ε4 = apolipoprotein E ε4; CSF = cerebrospinal fluid; Aβ = amyloid-β 1-42; T-tau = total tau; P-tau = tau phosphorylated at threonine 181; RCFT = Rey Complex Figure Test; JoLO = Judgment of Line Orientation; RAVLT = Rey Auditory Verbal Learning Test; TMTA = Trail Making Test Part A; TMTB = Trail Making Test Part B; GLM = general linear model.
[1]Statistic corresponds to W for Wilcoxon rank-sum tests, χ^2 for chi-square tests, and t for general linear models adjusted for age and sex
Significant at $^{***}P < 0.001$, false discovery rate corrected.

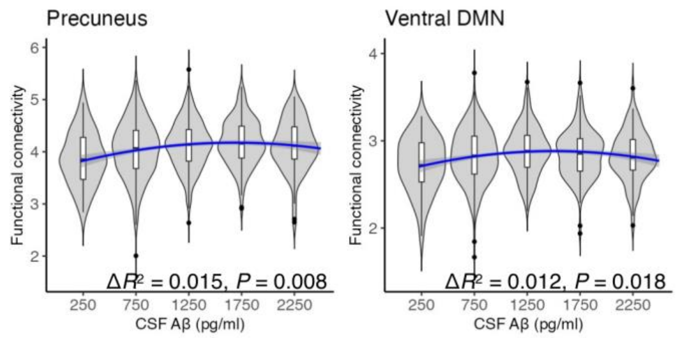

Supplementary Fig. S1. Violin plots illustrating non-linear associations between cerebrospinal fluid (CSF) amyloid-β 1-42 (Aβ) levels and functional connectivity in the precuneus network and ventral default mode network (DMN). The distributions of functional connectivity are shown across the range of CSF Aβ values, with fitted quadratic curves (blue curves) and 95% confidence intervals (shaded areas) overlaid. These plots highlight the inverted U-shaped relationships observed in the main analyses (see Fig. 2), providing a clearer visualization of the non-linear trends.

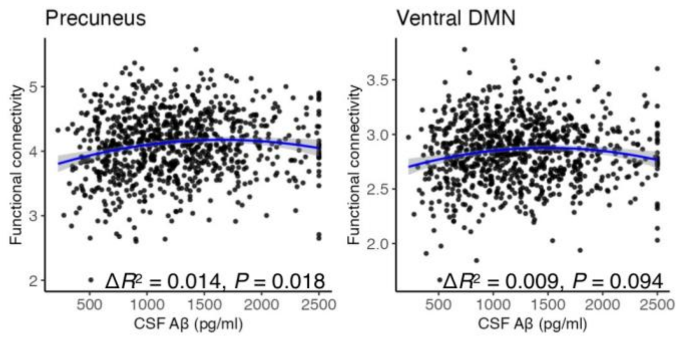

Supplementary Fig. S2. Non-linear associations between cerebrospinal fluid (CSF) amyloid-β 1-42 (Aβ) levels and functional connectivity in the precuneus network and ventral default mode network (DMN). Quadratic associations are shown by blue curves, with shaded areas representing 95% confidence intervals. Associations were evaluated using multiple regression adjusted for age, sex, education, and apolipoprotein E ε4, with *P* values corrected using the false discovery rate.

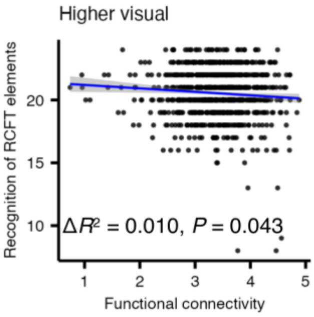

Supplementary Fig. S3. Association between higher visual network connectivity and the recognition of Rey Complex Figure Test (RCFT) elements. Stronger connectivity in the higher visual network was associated with poorer RCFT recognition performance (β = -0.101, 95% CI: -0.169 to -0.033, $\Delta R^2 = 0.010$, $P = 0.043$, FDR corrected). Linear association is shown by a blue regression line, with the shaded area representing 95% confidence interval. Association was evaluated using multiple regression adjusted for age and gender, and the *P* value was corrected using the false discovery rate.
